# Genotype and Ancestry Modulate Brain's DAT Availability in Healthy Humans

**DOI:** 10.1371/journal.pone.0022754

**Published:** 2011-08-03

**Authors:** Elena Shumay, John Chen, Joanna S. Fowler, Nora D. Volkow

**Affiliations:** 1 Brookhaven National Laboratory, Department of Medicine, Upton, New York, United States of America; 2 Department of Preventive Medicine, State University of New York, Stony Brook, New York, United States of America; 3 National Institute on Drug Abuse, National Institutes of Health, Bethesda, Maryland, United States of America; University of North Dakota, United States of America

## Abstract

The dopamine transporter (DAT) is a principal regulator of dopaminergic neurotransmission and its gene (the *SLC6A3*) is a strong biological candidate gene for various behavioral- and neurological disorders. Intense investigation of the link between the *SLC6A3* polymorphisms and behavioral phenotypes yielded inconsistent and even contradictory results. Reliance on objective brain phenotype measures, for example, those afforded by brain imaging, might critically improve detection of DAT genotype-phenotype association. Here, we tested the relationship between the DAT brain availability and the *SLC6A3* genotypes using an aggregate sample of 95 healthy participants of several imaging studies. These studies employed positron emission tomography (PET) with [^11^C]cocaine wherein the DAT availability was estimated as Bmax/Kd; while the genotype values were obtained on two repeat polymorphisms - 3-UTR- and intron 8- VNTRs.

The main findings are the following: 1) both polymorphisms analyzed as single genetic markers and in combination (haplotype) modulate DAT density in midbrain; 2) ethnic background and age influence the strength of these associations; and 3) age-related changes in DAT availability differ in the 3-UTR and intron8 – genotype groups.

## Introduction

Behavioral- and neurological disorders are highly heritable [1], [2], yet, identification of the specific genetic factors underlying such diseases is very challenging. Human brain function is inherently complex and highly redundant, hence, we expect that the effect of any single genetic variation would be small, and thus, difficult to ascertain. By enabling the detection of such small changes on the level of the brain, imaging technologies facilitate objective, quantifiable measures of neural- and biological processes, known as “intermediate phenotypes” [3] in contrast with the more ambiguous behavioral phenotyping.

Dopaminergic circuits are central to many key brain functions, i.e., memory, locomotion, reward mechanisms, motivation and cognition (see recent reviews [4] and [5]). Thus, it is not surprising that the disturbances in dopaminergic tone are implicated in a broad spectrum of neuropsychiatric disorders, including attention-deficit-hyperactivity disorder (ADHD) [6], Parkinson's disease (PD) [7], schizophrenia [8], and diseases of addiction [9]. Direct assessment of the dopamine (DA) system in the human brain can be achieved through the examination of post-mortem brain tissue or through brain imaging (i.e. positron emission tomography, or PET). Postmortem brain studies allow to measure the amount of protein- (receptors, transporters and enzymes) or the cognate mRNA, while PET imaging makes use of radiolabelled tracers to quantify components of the dopaminergic circuitry, such as the DA transporters (DATs) and receptors (D_2_R, D_1_R).

DAT is the main regulator of dopamine signaling in the brain; it recycles DA back into the presynaptic terminal, thus terminating synaptic firing. Methodological and experimental challenges limit our understanding of the biological mechanisms governing DAT activity and availability [10]. Thus, in imaging studies it is not always possible to determine whether decreases in DAT reflect the downregulation of DAT expression, or rather, decrements in the quantity of the DA terminals. To gain new insights into the DAT's regulatory network, we recently scrutinized the genomic features of the human *DAT1* gene (*SLC6A3*) and identified several unusual characteristics of this locus, including an unusual abundance of repeated elements [11].

Polymorphic repeats, such as the variable number of tandem repeats (VNTRs), occur frequently in brain-related genes and often are robustly linked to various behavioral phenotypes [12], [13]. The most studied polymorphism in the *SLC6A3* is a 40-bp VNTR located in the 3′-untranslated region of the gene (herein referred to as 3′UTR) which produces two major alleles with 9- and 10-repeats (viz., 9R and 10R). Its functional effect was investigated intensely in experimental settings yielding disparate findings : Thus, while Michelhaugh et al. showed that the 9R-repeat sequence enhanced the transcription of the reporter construct [14], Fuke et al. [15] reported the same effect but for the 10R-vector and Greenwood et al. [16] found that neither sequence affected the transcription rate. Similarly, analysis of this polymorphism with *in vivo* imaging gave inconsistent findings : Van Dyck and van de Giessen reported the higher availability of striatal DAT in 9R-allele carriers [17]; Heinz et al. [18] found reduced DAT density in 9R-carriers (9/10 heterozygotes) compared with 10R homozygotes and, finally, Martinez et al. [19] failed to detect any changes associated with the DAT1 VNTR. The two largest studies encompassing only healthy subjects (Van Dyck et al., −96 individuals [17] and van de Giessen et al., −79 individuals [20]) reported an increase in DAT availability associated with 9R allele.

Several variables might contribute to the conflicting results of imaging studies; the most apparent are: the heterogeneity of the populations under study, the selection of statistics, the technical characteristics of the scanners, the choice of radiotracers and the mathematical models used for image processing, analysis and quantification. Because of the technological complexity and the high cost associated with PET imaging a single study typically affords only a limited sample size; this, in turn, diminishes statistical power and limits the interpretation of the results.

It is unclear whether the 3′UTR VNTR exerts direct functional effect or it is a genetic marker for another variant in the same linkage disequilibrium (LD) block. For example, clinical genetic studies of ADHD [21] indicated that the 10R allele increases risks for the disease when combined with a particular variant of the VNTR that resides in the intron8 of the gene. This polymorphism (herein, intron8) produces two common alleles comprising 5- and 6 repeats of the 30 bp period (viz., 5R and 6R). The functional significance of this variant was confirmed *in vitro* [22], [23], but the single PET study published so far failed to associate this variation with DAT's brain measures [24].

Empirical evidence clearly demonstrated a pronounced effect of the individual's **ancestry** on the brain's dopaminergic measures [25] but, historically, with a few exceptions [25] population of most clinical-, molecular-, and neuroimaging- studies comprised individuals of European descent. We currently do not know the extent to which imaging results are transferable to other populations; hence, it is critical to establish their generalizability by studying diverse ethnic groups. Extensive differences in the allele frequencies and heterozygocity among different ethnic groups have been consistently reported for the *SLC6A3* locus [26], and these differences could manifest as different biological values of genomic factors [27].

**Age-related** decline in DA activity has been established based on *post-mortem*-[28], [29] and *in vivo* imaging studies [30]. The age-related decreases in DAT density vary among individuals thus suggesting that genetic variations might play a modulating role.

At Brookhaven Center for Translational Neuroimaging we have conducted several clinical PET studies to investigate DAT availability in a variety of diseases and conditions [31], [32], [33]. Here, we took advantage of the imaging database and generated an aggregate sample of healthy individuals that participated in different studies as controls. Using this heterogeneous sample that included individuals of different age, gender and ancestry we tested the relationships between the DAT brain endophenotype and two polymorphisms of the *SLC6A3* gene, the 3′-UTR- and the intron8 VNTRs either as single genetic markers or in combination, as a haplotype.

Building on previous knowledge, we developed the following **hypotheses:**

**Figure 1:**
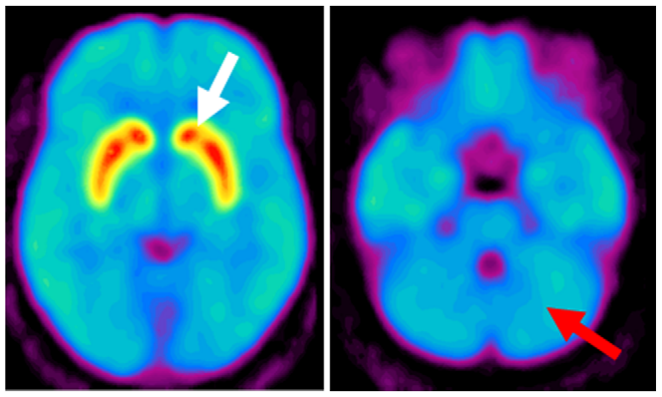
The DAT binding potential in the human brain. Brain images of dopamine transporter (DAT) availability obtained using [^11^C]cocaine at the levels where the striatum (left image) and cerebellum (right image) are located. A rainbow color scale is used to represent DAT availability (red>yellow>green>blue>purple. Images are averaged from brains of 20 healthy participants. Note the high DAT levels in striatum (white arrow) and the minimal levels in cerebellum (red arrow).

1. Since the dopaminergic neurons in different brain regions have distinct anatomical- and morphological-characteristics of [34], we hypothesized that the genetic variations might differentially contribute to the *SLC6A3* regulation, so variably affecting DAT density (as measured by PET) within striatal sub-regions (Figure 1).
2. Because the two VNTRs are located in different haplotype blocks, we hypothesized that the putative genotype-DAT brain phenotype associations for each genetic variant can be discreet. At the same time, considering the strong LD between the variants, it is possible that one VNTR would be a marker for the effect driven by the other VNTR.
3. We hypothesized that *ancestry* would modulate the genotype – brain phenotype relationships so that the significance of the associations ascertained in populations of European descent (CE) may be less in African-American populations (AA).
4. Based on the observations on *age-related* decline, we hypothesized that the *SLC6A3* polymorphisms may differentially affect this process. Considering the unusually high epigenetic sensitivity of the *SLC6A3* gene [11], and the notion that the density of DNA methylation increases with age genome-wide [35], we hypothesized that the effect of the genotype on measures of brain DAT would be more apparent in younger individuals.

## Materials and Methods

### Study Participants

The study was approved by the local Institutional Review Board (Committee on Research Involving Human Subjects, State University of New York at Stony Brook) and carried out at Brookhaven National Laboratory. Written informed consent was obtained from each subject after the study had been fully explained to them. Participants were paid for providing a genetic sample and for participating in the imaging study.

The study population consisted of 95 healthy adults that served as healthy controls in different brain imaging studies. Table 1 summarizes demographic characteristics of the sample which is representative of the local community (ethnicity is reported based on participants' self-assignment). All individuals were free of present or past history of a medical, psychiatric or neurological disease, including past or present histories of substance use disorders. Subjects were included in this study based on the availability of [^11^C]cocaine brain scans and a written informed consent documenting the individual's agreement to participate in a genetic study.

**Table 1:** Sample Demographics.

*Race, Ethnicity*	*N (%)*	*Sex*	*N (%)*	*Age* *(mean+/−SD)*	*Age* *(range)*
African-American (AA)	33 (38)	M	28 (85)	33.7+/−7.5	21.5–45.5
		F	5 (15)		
Caucasian (CE)	52 (56)	M	43 (83)	35.8+/−7	20.5–49.5
		F	9 (17)		
Others	10 (11)	M	7 (70)	30.2+/−5	22.2–40.3
		F	3 (30)		

Ethnic group “Others” included 5 Hispanics- and 5 individuals of more than one race.

### Positron Emission Tomography

Imaging data with [^11^C]cocaine associated with each genetic sample were retrieved from the imaging dataset of the BNL Brain Imaging Center. All PET scans used in the current study were performed on a Siemens, HR+ scanner. The procedures for subjects positioning and the scanning protocols were described previously [33]. The imaging results for the healthy subjects have been reported as part of the control data for comparison with specific mental states or disease conditions [33], [36], [37], [38], [39]. Similarly, the analytical approach for measuring DAT availability has been previously [40], [41]. Briefly: Regions of interest (ROI) including *caudate*, *putamen*, and *ventral striatum* in both hemispheres were drawn directly on an emission image that represented the sum of images obtained during 10–54 min scans. Right and left cerebellar regions were drawn in three planes, 1.0, 1.4 and 1.8 cm, above the canthomeatal line. ROIs were then projected onto the dynamic images to generate time activity curves for *striatum* and *cerebellum*. Average values for the basal ganglia and cerebellar regions were computed from three slices, and values from the two hemispheres were also averaged. The cerebellum, where DAT expression is almost negligible, was used as a reference region. The combination parameter Bmax/KD which is a measure of free transporter concentration (binding potential) was calculated as the ratio of the distribution volume in basal ganglia to that in cerebellum minus 1; it was obtained using a graphical analysis method without blood sampling technique for reversible systems [42].

### Genotyping

Genomic DNA was extracted from whole blood samples using Qiagen kit according to a protocol recommended by the manufacturer. Two tandem repeat polymorphisms located in the 3′ untranslated region of the *SLC6A3* (3′-UTR VNTR) and in the Intron8 (Intron8 VNTR) were genotyped. Genotyping was performed as described elsewhere: The 40-bp 3′UTR 40-bp VNTR – as described in [43] and intron8 30-bp VNTR – as detailed in [44]. The exact size of the amplified DNA fragments was established by using the QIAxcel system of a multi-capillary electrophoresis with high resolution cartridge (QIAGEN).

### Statistical Analysis

#### The DAT brain data were inspected for normality

Data on heterozygosity were obtained from ALFRED database (ALFRED, http://alfred.med.yale.edu/alfred/recordinfo.asp?condition=loci.locus_uid=%27LO000196Q) confirmed the common distribution of the investigated allelic variants in both, AA and CE populations (the observed heterozygosity in 3′UTR locus was 46% in the AA-, and 38% in the CE- populations: in the intron8 locus – 50% and 42%, respectively). The genotype distribution for both VNTR variants and its agreement with Hardy-Weinberg equilibrium (HWE) assumption were assessed using an online server - http://www.oege.org/software/hwe-mr-calc.shtml [45] .

#### Genotype-based classifications

The sample was divided on groups based on three-genotypes-classification. The group assignment for the 3-UTR genotype was according to three common genotypes (9R/9R vs 9R/10R vs 10R/10R). This partition excluded carriers of rare genotypes. All individuals of the sample carried the common alleles of the intron8 variant so that all subjects were assigned to the groups based on three genotypes- classification (5R/5R vs 5R/6R vs 6R/6R). Table 2 shows the genotype- and allele frequencies in populations.

**Table 2:** Genotype- and Allele- Frequencies in Sample Populations.

	*3′UTR*					*Intron8*				
	9/9	9/10	10/10	MAF (9R)	HWE (χ2)	5/5	5/6	6/6	MAF (5R)	HWE (χ2)
**AA**	2 (6%)	10 (30%)	18 (55%)	0.24	0.14	5 (16%)	22 (65%)	6 (19%)	0.33	3.69
**CE**	6 (10%)	25(47%)	20(43%)	0.26	0.19	1 (2%)	25 (48%)	26 (50%)	0.25	3.27
**others**	0	4 (40%)	6 (60%)	0.3	0.62	0	5 (50%)	5 (50%)	0.25	1.1

**Abbreviations:** AA-African-American (N = 33), CE-Caucasians (N = 52), others-(N = 10); MAF-minor allele frequency, HWE- Hardy-Weinberg equilibrium.

Three AA individuals (9%) and one CE (2%) carry rare 3-UTR alleles.

#### Haplotype assignment

Haplotypes were constructed for the two repeat polymorphisms, 3′ UTR and intron 8 VNTRs using an Expectation-Maximization (EM) algorithm [46]. Estimates of sample haplotype frequencies together with their standard deviations, a list of all pairs of possible haplotypes for each individual were derived using SAS PROC Haplotype (SAS Institute, Cary, NC). Haplotype frequencies were first estimated within each ethnic group; then among all subjects. The most probable phased genotype was assigned to each subject for further analysis.

Haplotype-based classification: Similarly to procedure used for the genotype-based partition, rare haplotypes were excluded, so only carriers of the common haplotypes were assigned to the groups based on combination of common alleles (four haplotypes- classification). The groups were: 10-6 (n = 27), 10-5 (n = 19), 9-5 (n = 37) and 9-6 (n = 8).

We used two approaches to address the anticipated confounding effects of ethnicity and age: 1) analyzing the sample as a whole, we controlled for population stratification by introducing ethnicity as a covariate; and 2) we performed separate within strata analyses for the two major ethnic groups, AA (n = 33), and CE (n = 52). In a similar manner, we controlled for age by 1) introducing it as a covariate, and 2) tested separately two age sub-groups divided by mean age (younger than 35 years, n = 50, range: 20.5–34 years; and 35 years and older, n = 45, range – 35–49.5 years).

In exploratory analyses we tested potential confounding effects of sex (77 subjects were males and 18 females) and smoking status (based on a self-report, 84 subjects were non-smokers, 4 -current smokers (less than 10 cigarettes per day) and 7 – former smokers). Fitting a GLM model showed low importance of the gender in respect to the DAT levels in genotype-based groups (*p* = .342) and lack of sex-genotype interaction (*p* = .685). Similarly, we did not detect differences in regional DAT density (Bmax/KD) between groups based on smoking status (smokers *vs.* former smokers *vs.* non-smokers (two-ways ANOVA: *caudate* - *F*(2, 92) = 0.709, *p* = .495).

Analysis of the relationships between the DAT brain phenotype and the *SLC6A3* genotypes was performed with ANCOVA (General Linear Model (GLM) where a regional Bmax/KD value was treated as dependable variable and genotype – as between-subjects factor. Age and race were introduced as covariates. The analyses were first done for the whole sample and then for the ethnic and age groups separately.

Because the brain data did not meet condition of sphericity, i.e., there were significant differences between the variances of differences (Maunchy's test statistic –*p*>0.001, Greenhouse-Geisser – 0.827) for ROIs, the three brain regions were analyzed independently.

A multiple regression model (age as a predictor of DAT values) was tested to verify effect of age and age-adjusted DAT availability in genotype groups. To enable comparison of the regression coefficients for the genotype groups and assessment of their differences on the basis of the *p*-value for the difference we constructed a regression model with a predictor that has two categories, viz., an interaction term between the each genotype (i.e., 9/9, 9/10 and10/10) and age.

All effects are reported at three conditions: the F value (*F*), the degrees of freedom (*df*) and the Sig. (*p* value). Partial eta-squared (*η_p_^2^* ^)^ is the measure of effect size. Effects reported as statistically significant at *p*<0.05.

The statistical tests were performed using SPSS software (version 11.5).

## Results

### Genotype composition

All individual samples of genomic DNA were successfully amplified using primers specific for the 3′UTR- and intron8- VNTR regions. We employed three different approaches to exclude genotyping errors and misclassification of the genotypes: 1) Resampling of the same individuals (the DNA samples were tested as duplicates, triplicates, or quadruplicates); 2) re-testing of the same samples using different primer set and optimized PCR amplification protocol; and, 3) sequencing randomly selected subsets of the PCR- amplified fragments (ABI 3100) (about every fourth DNA sample). All those tests produced the same results confirming the correct assignments of the genotype's categories.

In our sample, genotype frequencies for two VNTRs were inter-correlated (Pearson *χ^2^* test, *p<*0.001). The overall frequencies of the major alleles of the 3′UTR VNTR, the 9R- , and 10R-alleles were 24% and 69%, respectively, analogous to Vandenbergh's findings [47] and ALFRED database (30% vs 70%). The frequencies of the 5R- and 6R-alleles were 40% and 60%, while Genro reported a 27% frequency of the 5R allele [48]. According to the ALFRED database, allele frequencies for intron8 site are 50%/50% and 30%/70% in AA- and CE-populations, respectively. The actual genotype frequencies for both VNTRs in a whole sample were close to those expected from the Hardy-Weinberg equilibrium (HWE) assertion. Observed deviations from HWE might be explained by non-binary nature of both VNTRs, even though only common alleles were used for calculations.

**Figure 2:**
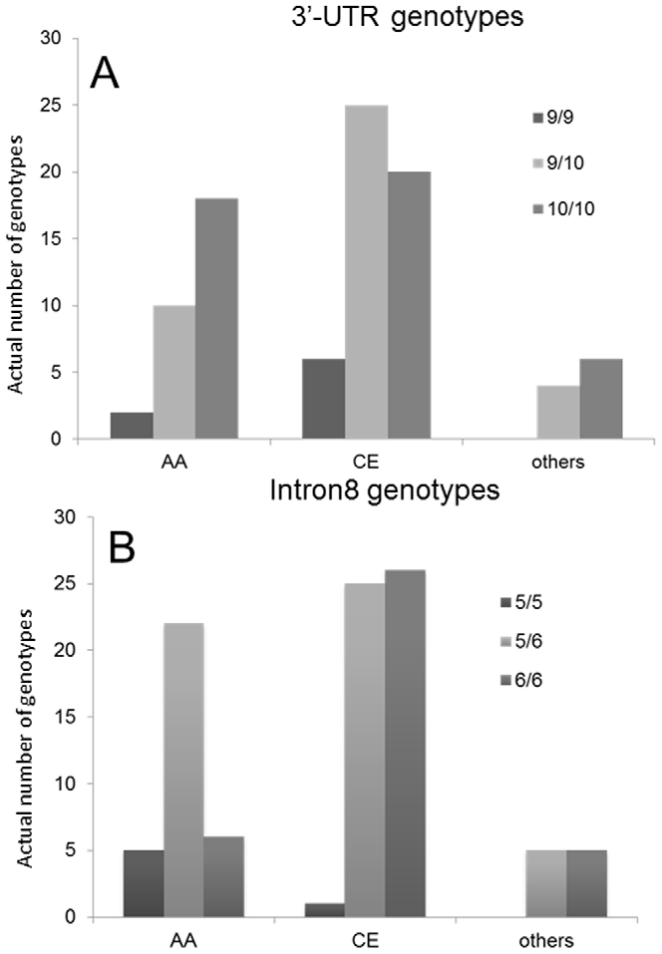
Genotype distribution in AA and CE populations. The height of the bars representing 3′-UTR genotypes (A) and intron8 genotypes (B) corresponds to occurrence of each genotype category (actual number of individuals) in groups comprising African-Americans (AA), Caucasians (CE) and other ethnicity (others).

Genotype- and allele- frequencies in ethnic groups markedly differ (Table 2 and Figure 2). Among AA, most individuals are homozygous (only 10 individuals are heterozygous 9R/10R carriers; the expected count under the HWE is 16). Conversely, more than expected CE individuals are heterozygous (observed 25, expected 20). More than expected AA individuals carry the heterozygous intron8 alleles (22 vs 15) while about half of the CE population is common homozygotes. These deviations from HWE in populations may indicate non-random sampling and small population size. The frequencies of intron8 genotypes in populations were significantly different: c^2^(4, (*N* = 95) = 12.5, *p* = 0.014. Only the most common genotypes for both VNTRs were observed in the group representing other ethnicities.

### PET imaging data

Our sample included participants from different research studies, thus, we took great care to confirm the reproducibility of the DAT measures. Excellent reproducibility of the imaging data was confirmed comparing the Bmax/KD values derived from the analyses of the [^11^C]cocaine scans obtained from the same individuals on two different occasions. This test-retest analysis (27 individual observations) revealed a robust link between the values of Bmax/KD from two different scans performed on two different days and time between the scans varying from 2 month to 3 years (i.e., *caudate*: *t* = −6.202; df = 21; *p<*0.001).

Exploratory analysis of the entire brain imaging dataset confirmed that the data were normally distributed (visual assessment of the QQ normal probability plots). The DAT availability notably differed among individuals, especially in the *ventral striatum*, but the skewness and kurtosis of the variables satisfied the assumption of normality, allowing analysis of non-transformed values. DAT availability (Bmax/KD) averaged 0.77 (*s* = 0.13) in *caudate*, 0.93 (*s* = 0.13) in *putamen* and 0.81 (*s* = 0.17) in *ventral striatum*.

### Genotype impact on the brain measures

**Figure 3:**
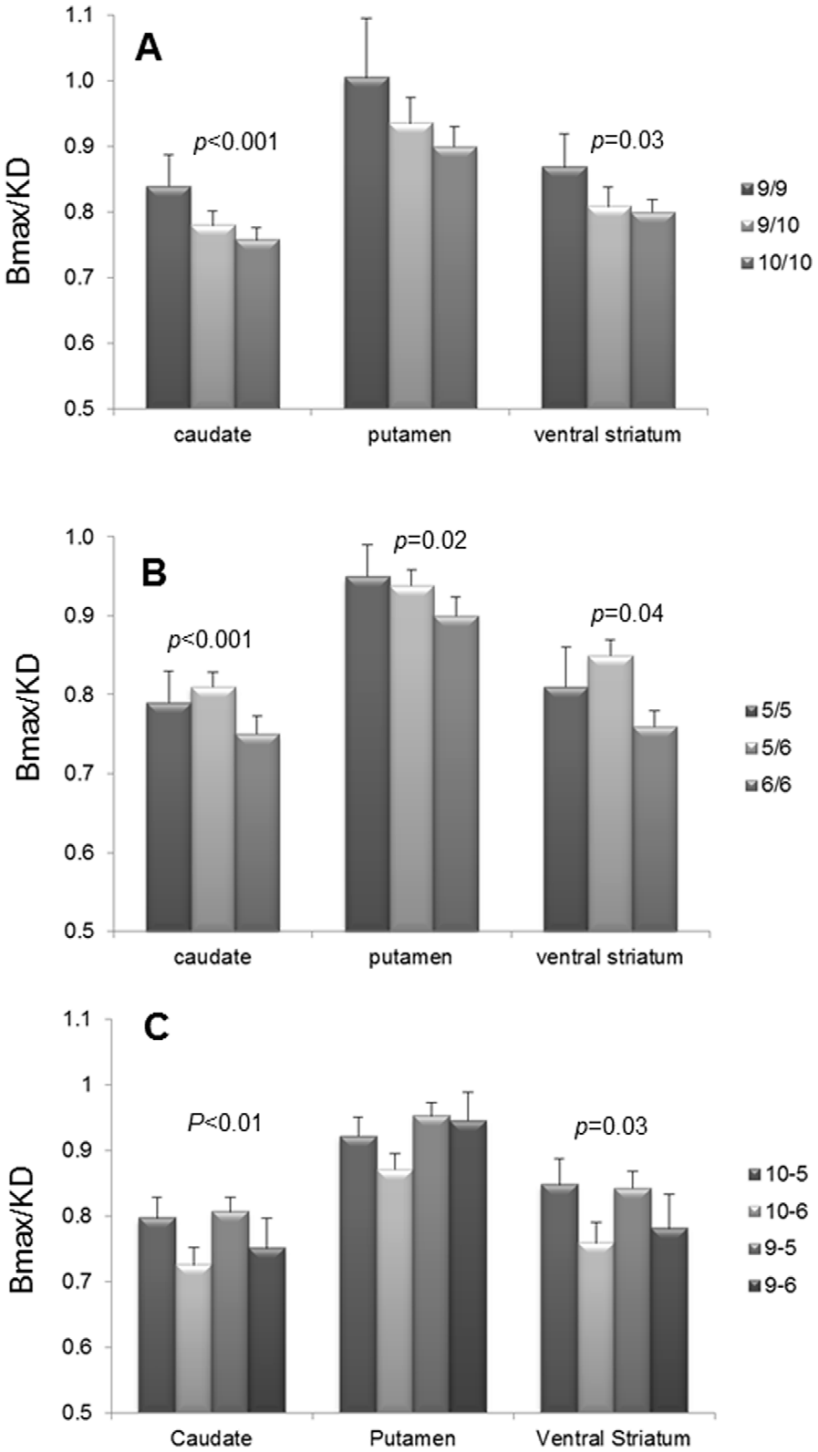
Measures of DAT availability (Bmax/KD) in genotype groups. Bars correspond to mean DAT availability and standard errors of means in 3′-UTR-genotype (A), intron8-genotype (B) and haplotype groups (C) in *caudate*, *putamen* and *ventral striatum*. Significant differences between the mean values are indicated (p<0.05). Bmax/KD values (Y-axis) correspond to DAT binding potential.

The differences between 3′-UTR (panel A) and intron8 (panel B) genotype groups in region-specific DAT measures are shown in Figure 3; statistical values of the analyses are summarized in Table 3. The overall relationship between the 3-UTR genotype and DATs levels was statistically significant in *caudate (F*(5,81) = 5.9, *p<*0.001, *η_p_^2^ = *0.25) and *ventral striatum* (*F*(5,77) = 3, *p = *0.03, *η_p_^2^ = *0.16). Main effect of the 3′UTR genotype and its interaction with age were not significant. The overall relationship between intron8 genotype and DAT levels in all three regions was significant: *caudate* – *F*(5,89) = 6.2, *p*<0.001, *η_p_^2^ = *0.3), *putamen* F(5,89) = 2.4, *p* = 0.02, *η_p_^2^ = *0.2) and *ventral striatum* – *F*(5,81) = 2.5, *p* = 0.04, *η_p_^2^ = *0.22). Main effect of the intron8 genotype was significant in *caudate*: *F*(5,89) = 3.1, *p* = .04, η_p_^2^ = 0.06 and in *putamen* - *F*(5,89) = 3.6, *p* = .03, *η_p_* ^2^ = 0.07. The interactions between the intron8 genotype and age also were significant in *caudate*: *F*(5,89) = 3.1, *p* = .04, *η_p_* ^2^ = 0.07 and in *putamen* - *F*(5,89) = 3.5, *p* = .04, *η_p_* ^2^ = 0.07.

**Table 3:** Relationships between the *SLC6A3* variations and regional DAT measures.

*Variant*	*ROI*	*df*	*F*	*p*
**3′UTR**	*Caudate*	5,81	5.9	<0.001*
	*Putamen*	5,81	2.1	NS
	*Ventral Str*	5,77	3.0	0.03*
**Intron8**	*Caudate*	5,89	6.2	<.001*
	*Putamen*	5,89	2.4	0.02*
	*Ventral Str*	5,81	2.5	0.04*
**Haplotype**	*Caudate*	5.85	2.6	<0.01*
	*Putamen*	5.85	1.7	NS
	*Ventral Str*	5,81	2.6	0.03*

**Abbreviations**: ROI – region of interest; Ventral Str. – ventral striatum.

Significance level for overall effect of the genotypes (three-genotypes classification) and haplotypes (four haplotypes classification) was set at *p*<0.05 (ANCOVA); values<0.05 are marked with*.

### Haplotype-based analysis

Mean DAT availability in haplotype groups is shown in Figure 3C. The overall relationship between regional DAT availability and haplotype was significant in *caudate*: *F*(5,85) = 2.6, *p*<0.01, *η_p_* ^2^ = 0.2 and in *ventral striatum*: *F*(5,78) = 2.6, *p* = 0.03, *η_p_* ^2^ = 0.14). Main effect of the haplotype and its interaction with age were not significant.

### Influence of ethnicity on DAT availability - genotype relationships

Apparent differences between the ethnicity-based groups in the frequency of the DAT alleles and heterozygocity (Figure 2) raised the question of whether the associations between the genotype and brain DAT measures detected in the sample as a whole would hold in stratified populations. To clarify this, we split the sample based on ethnicity and analyzed separately the AA (n = 33) - and the CE (n = 50) subgroups. The relationships between the 3-UTR genotype and DAT measures were significant in the CE subgroup in all three ROIs (*caudate - F*(5,43) = 5.67, *p*<0.001); *putamen* – *F*(5,43) = 3.17, *p* = 0.015; *ventral striatum* – *F*(5,38) = 3.18, *p* = 0.015) but not in the AA subgroup. Effect of the intron8 genotype was similar in both subgroups.

### Age effect on Genotype-DAT endophenotype associations

To test our hypothesis that the putative genotype effect on DAT measures would be stronger in younger individuals, we tested two age sub-groups separately (two -way ANOVA). An association between the 3′UTR genotype and DAT availability in younger individuals (n = 50) was significant across all regions, viz., *caudate* (*F(2, 47)* = 3.74, *p* = 0.03), *putamen* (*F(2, 47)* = 6.28, *p*<0.001) and *ventral striatum* (*F(2,44)* = 3.38, *p* = 0.04). In contrast, in the group of older individuals (n = 45) the association was not significant. Similarly, the intron8 genotype was strongly associated with DAT availability in younger individuals (*caudate*: *F*(2,47) = 7.05, *p<*0.001; *putamen*: *F(2,44)* = 5.36, *p* = 0.01) but not in older ones.

### Genotype effect on the age-related decline in DAT density

ROI-based regression analysis demonstrated a strong (negative) relationship between DAT availability and age in *caudate (F*(1, 93) = 20.5, *p*<0.001) and *ventral striatum* (*F*(1,85) = 6.2, *p* = 0.015), but not in *putamen* (*F*(1,93) = 2.7, *p* = 0.13).

**Figure 4:**
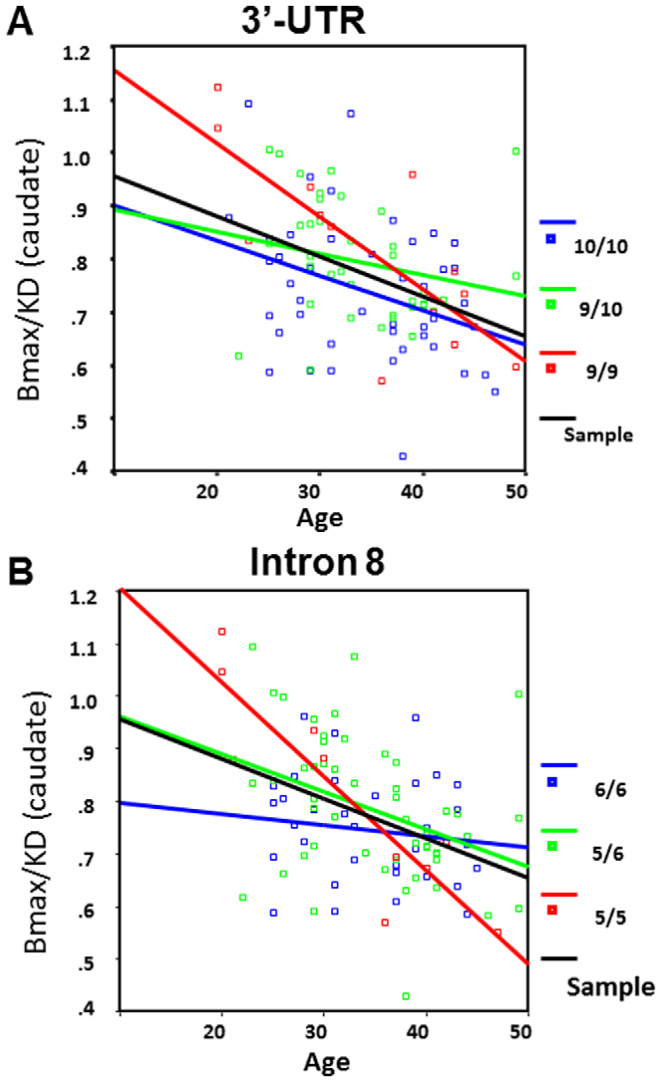
Age-related changes in the DAT binding potential. Scatterplots show the regression between the measures of DAT availability in striatum (Y-axis) and age (X-axis) in 3′-UTR- (A) and intron8-(B) genotype-based groups. Black regression line – all subjects of the sample, colored lines – genotype-based subgroups.

To test whether the regression coefficients of age predicting DAT availability would differ between the genotype groups, we separately considered the data for each group (*caudate*). Table 4 shows the parameter estimates (coefficients) for the genotype groups. The results seemingly suggest that age is a stronger predictor of Bmax/KD value in the 9R/9R (b = −.781)- and the 5R/5R (b = −.7)- groups. Graphical assessment of the regression model fit (Figure 4) demonstrated marked differences between the gradients of the regression lines for the 3′UTR genotype groups (Figure 4A) and the intron8 genotype groups (Figure 4B).

**Table 4:** Genotype - DAT measures relationships in populations.

	ROI	Population	*F*	*df*	*p*	*η_p_^2^*
**3′-UTR**	*Caudate*	AA	4.15	5,26	<0.01*	0.4
		CE	5.67	5,44	<0.001*	0.3
	*Putamen*	AA	0.71	5,26	*0.621*	0.25
		CE	3.17	5,44	0.015*	0.2
	*Vent.Str*	AA	0.95	5,22	*0.474*	0.1
		CE	3.18	5,40	0.015*	0.26
**Intron8**	*Caudate*	AA	3.50	5,28	0.015*	0.4
		CE	3.42	4,47	0.015*	0.2
	*Putamen*	AA	0.44	5,28	*0.817*	0.08
		CE	1.81	4,47	*0.142*	0.13
	*Vent. Str*	AA	0.88	5,24	*0.513*	0.19
		CE	2.42	4,46	*0.061*	0.17

**Abbreviations**: ROI – region of interest; Vent. Str. – ventral striatum; AA- African-Americans, CE- Caucasians,

Significant values are indicated with *.

To better characterize the decline in DAT availability with age and to reveal putative effects of the genotype on this process we applied regression analysis separately to each genotype-based subgroup. Table 5 illustrates the acceptability of the model for different ROIs. To compare the regression coefficients among the three genotype groups and to assess their differences we tested the null hypothesis – H_0_: B_1_ = B_2_ = B_3_ where B_(n)_ is the regression for each of the genotype groups. For this analysis we created new variables (a code for each genotype and an interaction term- genotype *times* age) and used these as predictors in the regression equation. The results of the analysis (*F*(2) = 5.4, *p*<0.001) showed that the null hypothesis can be rejected based on significant differences between coefficients.

**Table 5:** Regression coefficients of DAT availability and age in genotype groups.

3′-UTR				Intron8			
*Genotype*	*b*	*t*	*p*	*Genotype*	*b*	*t*	*p*
9/9	0.79	−4.2	0.002*	5/5	0.7	−2	0.12
9/10	0.2	−1.3	0.2	5/6	0.34	−2.5	0.015*
10/10	0.43	−3.1	0.003*	6/6	0.49	−3.4	0.01*

**Abbreviations:** *b*-(beta weights), standardized coefficient; *p*-significance, values<0.05 are marked with*; *t*-score (t-statistic is the coefficient divided by its standard error), significant at ItI>2.

## Discussion

Here, we evaluated the relationships between two DAT1 polymorphisms (3′-UTR- and intron8 VNTRs) and DAT brain availability in 95 healthy individuals and found that 1) both polymorphisms are significantly associated with striatal DAT density as single genetic markers and in combination, as a haplotype; and, 2) genetic background and age influence the strength of these associations.

Our investigation replicated reports from the other groups on associations between the 3′UTR genotype and DAT endophenotype [18]–[21]. However, there are important distinctions between our study and the previous ones. Whilst the other studies used allele-based classification of the genetic variances comparing 9R/10R- and 10R/10R-carriers in an ethnically homogeneous population (white Europeans), we used three-genotype classifications studying ethnically heterogeneous sample. Even though population genotypes frequencies are un-equal and, consequently, in our sample only 8 out of 95 individuals were homozygous carriers of 9R allele, we choose this classification because genotype-based analysis is preferable for studying mixed population when ethnic sub-populations differ in allele-frequency distribution and homozygosity [49], (Table 2 and Figure 2). Further, this methodology was dictated by unknown genetic model for the *SLC6A3* locus.

Our approach allowed us to confirm previously reported higher DAT levels associated with presence of 9R allele [17], [50], [20], and to go beyond and contribute novel information on allele effect in the genotype context. We found that 9R allele has an additive effect on DAT density, such that DAT measures incrementally increase as a factor of the 9R allele number (0 vs 1 vs 2, i.e., 10R/10R vs 9R/10R vs 9R/9R) (Figure 3).

Separate analyses of the two VNTR groups reveal that both genotypes affect DAT brain measures. The overall relationships between the intron8 genotype and Bmax/KD are statistically-significant in all striatal sub-regions (Figure 3). This is the first time that an association between the intron8 VNTR genotype and DAT availability has been reported. A prior imaging study [24] that measured DAT availability in 27 young males using SPECT with TRODAT-1 failed to detect an association between DAT density and the intron8 genotype. This discrepancy might be attributed to differences in sample sizes (our sample was larger, with greater power to detect the genotype effects), methodological- and instrumentation- differences (Instruments: SPECT vs PET; Radiotracers: TRODAT-1 vs [^11^C]cocaine) and also differences between the populations studied (inhabitants of São Paulo, Brazil vs inhabitants of Long Island, New York).

Analysis of the haplotype groups provided new information that cannot be inferred from the testing single genetic markers. We demonstrated haplotype effect on DAT availability (Figure 3C), and found that the effect of a particular haplotype is differentially modulated by a brain sub-region. For example, while the smallest Bmax/KD values are consistently seen in 10-6 haplotype group, an effect of the 9-6 haplotype is notably changing from region to region (compare the 9-6 bars in *caudate* and *putamen*). These results suggest that higher DAT levels seems to associate with presence of 5R allele (compare 10-5 with 10-6 and 9-5 with 9-6), thus underscoring a potential functional effect of intron8 variant. Indeed, this observation is very preliminary, yet, if replicated, it might help to explain inconsistencies of the previous finding in respect to 3′UTR polymorphism.

**Figure 5:**
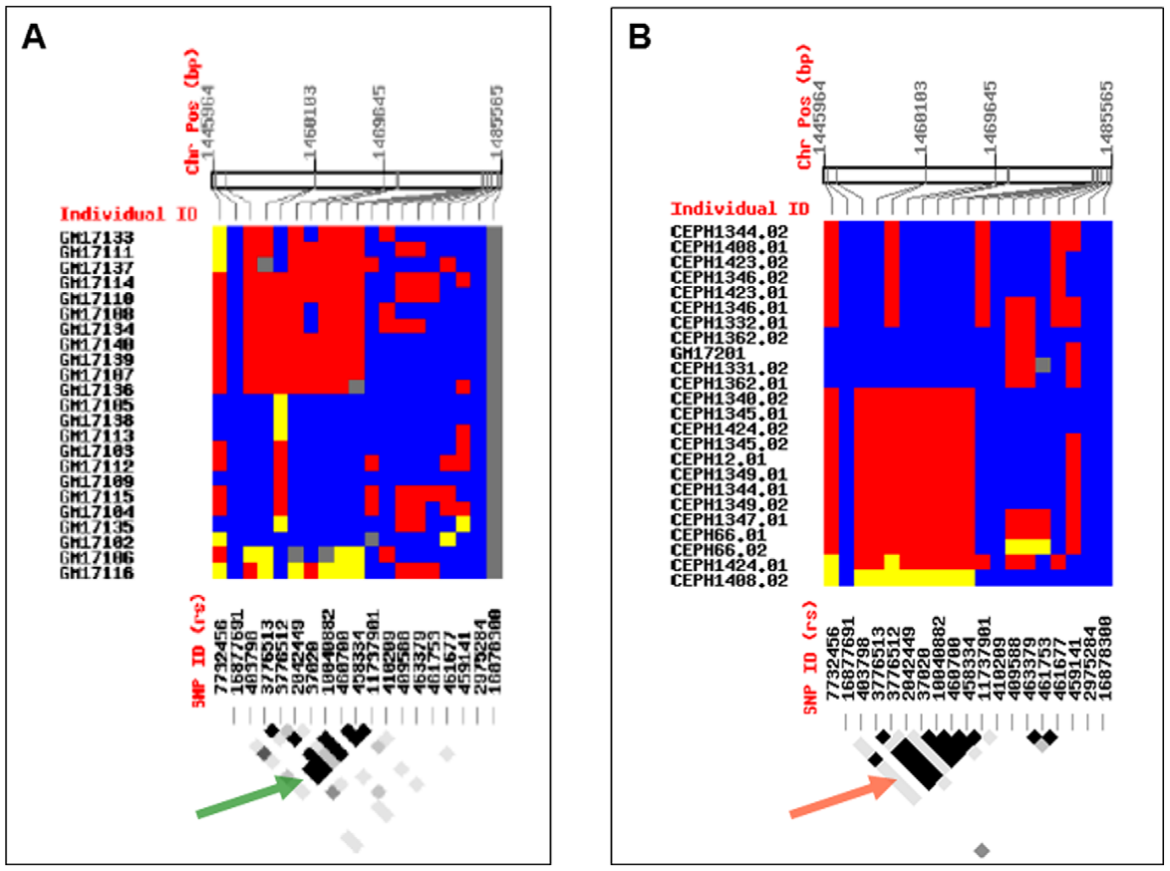
Haplotype structure of the *SLC6A3* 3′UTR locus in African-American- and Caucasian- populations. Graphical representation of the haplotype structure of the 3′-UTR region: African-American population - Panel A, top; Caucasian population - Panel B, top. Color scheme: blue – homozygotes, common allele, yellow – homozygotes, rare allele, red – heterozygotes, and grey – undetermined. Data are retrieved using Genome Variation Server (http://gvs.gs.washington.edu/GVS). Bottom of the panels illustrates corresponding haplotype maps: Confidence Bounds Color scheme: Strong evidence of LD – dark grey, uninformative- light grey and white color indicates strong evidence of recombination. More of dark grey in CE (red arrow) is indicative of the presence of LD block which is population-specific, whereas the light grey and the white color are predominant in the haplotype map of AA population (green arrow), indicate high recombination rate.

**Figure 6:**
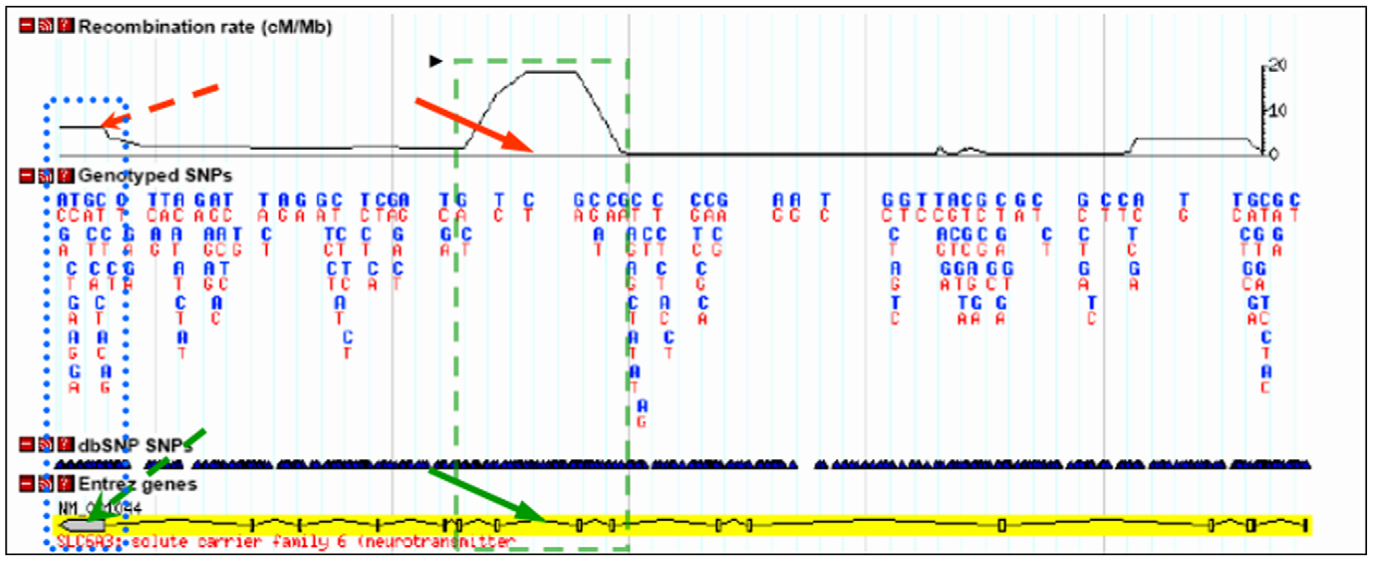
Increased recombination rate in the VNTR-encompassing regions of the *SLC6A3* locus. Both VNTR regions are within the recombination Hotspots indicated by red arrows (HapMap Data Release 27 PhaseII+III, Feb09, on NCBI B36 assembly). The green slashed box encloses a recombination hot spot (upper panel) that is projected to the intron8 (green arrow, bottom panel); the blue dotted box encloses a recombination hot spot (upper panel) mapped to the 3′UTR – region (green arrow).

Despite the numerous observations on marked differences between populations in the frequencies of the *SLC6A3* allelic variants across populations [51], [52] [53], there is a dearth of empirical data on the impact of ancestry on brain DA functions. Indeed, the *SLC6A3* locus was denoted as having the highest between-population differentiation (0.009<or = F_ST_<or = 0.012 [54] and, as illustrated in Figure 5, LD (linkage disequilibrium) plots for AA- and CE-populations are markedly dissimilar (HapMap project). Both polymorphisms reside within genomic hotspots (Figure 6) so that the mutagenic properties and high recombination rate of those regions as well as the forces of natural selection, all might impact variations in this locus [55]. However, most DAT imaging studies focus on European populations and thus their generalizability to other ethnic groups needs to be tested. Here we studied a diverse sample to gain insight on the connection between the genotype and its phenotypic correlates across a particular population, as, for instance, was discovered in the genetic associations for multiple sclerosis [56].

Our analysis showed that an association between DAT availability and the *SLC6A3* genotype is robust to population stratification, whereas subsequent analyses of AA and CE sub-populations revealed a differential impact of the genotype in these subgroups, especially notable in *putamen* and *ventral striatum* (Table 4). For example, the 3′UTR genotype – Bmax/KD putamen relationships were significant in CE (*p* = 0.02), but not in AA (*p* = 0.62). The difference in effect of the genetic variances might contribute to the differences in the rate of brain uptake of cocaine peaks between AA- and CE-populations reported previously by our group [25].

In light of this reasoning, it seems to us that selection of the participants by matching cases and controls by race may be insufficient, and that the influence of ethnic background on genotype- endophenotype associations can bias the findings. In fact, epidemiological data readily confirmed this, demonstrating that the 10R/10R genotype appears to be a marker for ADHD in CE populations [57], but not in AA populations [58]. In addition, because populations with more proximal affiliation with Africa have overall higher genetic heterogeneity and more genetic variations, it is possible that in non-Europeans, the other genetic variances, for example, polymorphisms in the 5′-*SLC6A3* [59], [60], [61] can offset the functional effect of the 3′ –UTR and intron8 VNTRs.

A substantial body of evidence clearly demonstrates the effects of age on DAT levels in the brain [29], [30]. Here we detected differences between the genotype-based subgroups in age-DAT availability relationships. The selective nature of the decline in DAT availability in genotype groups is evident in the non-parallel regression lines for the subgroups (Figure 3). This pattern is indicative of possible age-DAT levels interactions [62] that may act only in some genotype groups. Specifically, the DAT decline with age is less noticeable in the group of 6R/6R (intron8)- and 9R/10R (3′UTR) carriers. In contrast, it is most precipitous in carriers of the 9R/9R genotype (3-UTR genotype-based groups) and in the homozygous carriers of the 5R allele (intron8 genotype groups). Intriguingly, this rapid drop in DAT availability with age coincides with a higher DAT density at a younger age. We consider two possible explanations of these results: Either the 9R/9R and 5R/5R genotypes are predisposing to a greater loss of DAT with age, or the combinations of the DAT alleles (i.e., 9R/10R and 6R/6R) are protective against age-induced DAT loss. The “antagonistic pleiotropy” concept suggested that several modalities of gene action with a potential to modulate aging [63], wherein “good alleles” are often inappropriately down-regulated in late life [64]. Conversely, a “paradoxical antagonistic pleiotropy” postulates that the negative effect of “bad” or “risk” alleles early in life, reverses to “good” late effects [64], thus explaining that the relatively low DAT availability at 6R/6R homozygous remains seemingly unchanged over the years so that the DAT measures in older 6R/6R carriers are higher than in carriers of other genotypes. In this regards, it is of interest that the 9R/9R genotype was previously linked to increased risk for Parkinson disease (PD) (OR 1.8) [65]. PD pathogenesis is associated with DATs loss; thus, our finding that the 9R homozygous carriers have the fastest age-induced decline in DAT may be related to their higher vulnerability for PD. However, because of the small samples sizes in the genotype subgroups (8 and 6, for 6R/6R and 9R/10R respectively), our results need to considered as preliminary and requiring replication.

The functional effects of the 3′UTR VNTR have been widely investigated (review [66]), but only few studies have focused on the intron8 variant. Even though the functional effect of the latter was demonstrated *in vitro* [23], it is not sufficient to make inference on its biological impact *in vivo*. Because intronic sequences are spliced-out and do not affect the protein product of the gene, the question of how this polymorphism modulates the amount of DAT protein in the brain remains open. We recently elaborated on the possible functional impact of variations in the *SLC6A3* non-coding sequences [11]. Here, we just note that repeated sequences confer elevated level of recombination, hence they are considered mutation hotspots [67] and often create functional genetic variants that affect susceptibility to common diseases [68] Indeed, there is a recombination peak near the intron8 VNTR (Figure 6).

### Methodological Considerations and Study limitations

Because our sample represented healthy individuals, we were able to investigate effect of the DAT1 polymorphism on the brain's DAT levels directly, without accounting for the changes associated with disease state. Strengths of this study also include consistency of the imaging, i.e., imaging modality, instrumentation and image processing, and genotype ascertainment with several independent methods. However, the findings of the study have to be considered in a context of certain limitations. *First,* the DAT measures used in this analysis were obtained only for striatal regions where DAT levels are high. In order to evaluate sparse DAT expression in cortical brain regions, a radiotracer with higher affinity than [^11^C]cocaine would be required. In addition, the use of advanced image-processing techniques may afford more objective definition of the ROIs. *Second,* our genotyping tests did not include other functional polymorphisms in the *SLC6A3* gene, such as SNPs rs265211 and rs2937639 [20] and novel repeat variations and thus, we cannot exclude influence of intra-locus interactions and non-allelic complementation on DAT phenotype. *Third,* even combined sample was not large enough to enable testing the genomic impact on age-related DAT changes in ethnic groups separately. Given inter-correlation between the two polymorphisms and three brain regions, here we are reporting nominal significant findings, noting that these results are in need for replication. *Fourth,* even though our sample contained carriers of so-called “rare” alleles, for statistical reasons, we excluded these individuals from the analyses of 3′UTR genotype. Nonetheless, we believe that studying rare variants is important and can further our understanding of the *SLC6A3* genetic architecture. *Fifth,* our demographic information was obtained via interviews, where race and ethnicity categories were self-assigned by participants. Self-assignment is reliable [69] and commonly used in clinical research, but objective tests for ancestry, such as ancestry informative markers (AIMs) [70] afford precise categorization.

**In summary**, our analysis yielded several new findings, offering new insights in the genetic regulation of the DAT in the human brain and showed that these relationships are modulated by age and ancestry. This knowledge may help us better understand the complex interaction between genes and environment and its contribution to neuropsychiatric diseases.
